# The relationship between the professional, social, and political experience and leadership style of mayors and organisational culture in local government. Empirical evidence from Poland

**DOI:** 10.1371/journal.pone.0260647

**Published:** 2021-12-01

**Authors:** Aldona Podgórniak-Krzykacz

**Affiliations:** Department of Labour and Social Policy, Faculty of Economics and Sociology, University of Lodz, Lodz, Poland; Universiti Pertahanan Nasional Malaysia, MALAYSIA

## Abstract

This paper aims to identify the organisational culture profiles of Polish municipalities and examine the influence of the professional, social and political experience and place-based leadership style of mayors on municipalities’ organisational culture profiles. The Organisational Culture Assessment Instrument was selected due to its suitability in assessing the organisation’s underlying culture. In the study, 917 mayors of municipalities in Poland, completing an on-line questionnaire. It was found that most of the Polish municipalities’ organisational culture is characterised by a clan type which is reflected in how employees are managed, how the organisation is held together, and how the organisation’s strategy is defined. The leadership style and the organisation’s success are hierarchy-focused, while the dominant characteristic is market type. The ANOVA and UNIANOVA analysis results suggest that the type of organisational culture depends on the type of municipality. The clan culture is dominant in rural municipalities. In urban municipalities, market culture and adhocracy are stronger than in rural municipalities, while clan culture is weaker. There is also an association between the dominant type of organisational culture and the mayors’ work experience in local administration and their membership in an NGO. The length of the mayor’s seniority in local government administration differentiates the importance of hierarchy culture, while his experience in the NGO sector strengthens the clan characteristics of the organisational culture of the office he heads. These findings provide important implications for the initiation and implementation of cultural change in local government administration and cooperation projects and local experiments. A cultural change is difficult to implement, and a change of mayor is not enough to initiate it. It requires planning and management. Cultural change may contribute to the increase of municipalities’ activity in cooperation’s projects and experiments. There is a need for more research on this topic to determine to what extent the organisational culture supports local cooperation projects.

## Introduction

Organisational culture is an important determinant for collaborative, citizen-centred governance and co-production of public services [1, 2]. A study about Dutch municipalities found that they organise citizen engagement in this way, which, to some extent, reflects the most preferred organisational culture in the municipality [3]. In turn, other authors have confirmed in their studies that the drive for innovation as a cultural norm determines a strategic change in local government [4] or engagement in collaboration [5, 6]. The importance of organisational culture in the hybrid coordination of city organisations is also considered by Leixnering et al. [7], and they prove that cultural coordination, rooted in the network mode, complements structural coordination, which is associated with hierarchy and market. The cultural mechanisms relieve structural coordination while structural mechanisms provide the shadow in which cultural coordination thrives.

The article aims to diagnose the types of organisational culture in Polish municipalities. The survey on this topic was carried out in 917 Polish municipalities. In the article, I seek answers to the following questions: (1) Do municipalities with different types and administration sizes differ in terms of organisational culture? (2) Is there an association between the type of organisational culture of municipal administration and the professional, social, and political experience of mayors acting as managers of local administration? (3) Is there an association between the municipal administration organisational culture and the mayors’ preferred place leadership style? The article presents the first research results on the importance of individual factors (professional, social, and political experience of mayors and preferred municipal leadership style) for the organisational culture of municipalities. In the study, the organisational-administrative factors such as municipality type and administration size are control variables.

The cultural profile of Polish municipalities was diagnosed using The Organisational Culture Assessment Instrument (OCAI) [8]. The OCAI questionnaire was completed by mayors, who are the managers of municipal offices in Poland, which allowed us to obtain a holistic view of the organisational culture of municipal offices. The OCAI is the most used, actual, and useful framework for defining organisational culture types. It is validated in a lot of research [8–11] that confirm the methodological and conceptual validity of the OCAI tool. The OCAI provides a diagnostic assessment of the existing organisational culture by examining core values, shared assumptions, and common approaches to working. It is based on the Competing Values Framework (CVF), considering the dimensions: (1) flexibility and discretion versus stability and control, (2) internal focus and integration versus external focus and differentiation. This conceptualisation results in four culture types, namely, market, adhocracy, clan, and hierarchy. This framework has proved to be useful for understanding the impact of managerial values on leadership in local government [12].

Many instruments for exploring organisational culture exist [13]. The OCAI was used in this study because it assesses the essential culture dimensions, namely, culture type, strength, and congruence [14]. It enables the assessment of organisational culture more comprehensively than other Organisational Culture Measurement Instruments [15] (such as, e.g., Core Employee Opinion Questionnaire [16], Corporate Culture Questionnaire [17]). Furthermore, compared with instruments such as Organisational Culture Survey [18] or Hofstede’s Organisational Culture Questionnaire [19], the OCAI has better validity and reliability [15]. Speed and ease of assessment are also important arguments for its use. Tool developers Cameron and Quinn point to its versatility and applicability to both private and public organisations [8]. In previous studies, the OCAI has been used to diagnose the organisational culture of both local governments [3, 20, 21] and their organisational units [22].

The article is structured as follows. The next section reviews the existing research results on the diagnosis of types of organisational cultures in local governments and the existence of a relationship between organisational culture and the professional experience of public managers and the style of municipal management. On this basis, hypotheses were formulated. Section 3 presents analyses of variances of the survey results and the hypotheses testing. The paper ends with a discussion and conclusion, presenting the study’s implications and avenues for future research.

## Literature review

### Organisational culture in local government

Several different categorisations of organisational culture have been established. The literature on organisational culture in public sector organisations classifies it according to four types: hierarchy, clan (group culture), adhocracy (developmental model), and market culture (rational model) [14, 23, 24]. Features typical of a clan culture are teamwork, consensus, human resource development and involvement, and participation. An adhocracy culture emphasises innovation, risk-taking, change, agility, flexibility, and a market culture emphasises clear goals, goal attainment, productivity and efficiency. A hierarchy culture emphasises a formalised and structured work environment, centralised decision making and authority, and values stability and uniformity.

Numerous studies show that public organisations exhibit characteristics typical of bureaucratic organisational culture such as authoritarianism, formalisation, focus on procedures, strong control and aversion to change [23, 25, 26]. Moreover, analyses of local governments functioning in different countries, despite different administrative cultures, political contexts, directions and pace of public management reforms, confirm numerous features of hierarchical culture in municipalities. However, features of clan culture are also strongly manifested in municipalities [3, 4, 27, 28]. Based on analyses of organisational culture in selected Polish local government units, similar conclusions about the dominance of hierarchy and clan culture were formulated [20, 21, 29, 30]. Polish rural municipalities differ from urban municipalities in terms of the dominant culture type. In rural municipalities, clan culture features prevail over hierarchical ones, while in urban municipalities, it is the other way round [20].

Research suggests a need to change organisational culture in local government [31, 32]. Managers of public organisations usually prefer cultural characteristics that are more external and less control focused, which is typical for adhocracy culture, and more emphasised human relations values, which characterise clan culture [26]. Both the prevalence and the strong preference for clan culture in public administration are explained by researchers in that the clan characteristics of administration are due to long careers in the same administration and the informal (social) ties that exist between individual employees [33]. According to Martin and Kloot [27], the market reforms of New Public Management aimed to make public organisations more business-like and market-oriented by adopting the management techniques and tools of private sector organisations [34], have not changed the clan organisational culture in Australian municipalities. These researchers explain this by the desire of managers to preserve their existing culture to maintain a sense of comfort in a period of constant change. The prevalence and persistence of clan culture is, in their view, also an effect of the long employment and experience of local government managers built up in the public sector, as well as practices of working with the local community. Furthermore, the difficulties in changing organisational culture result in the emphasis on functionally oriented and traditionally hierarchical structures and control systems that are especially anchored in the public sector [35]. Orr and Vince [36] argue that local authorities are not only considered to tend to "segmentalist" cultures, but the cultures themselves are also deeply entrenched within the organisation and therefore difficult to influence.

### Impact of the experience of directly elected mayors on local policymaking and organisational culture of municipal administration

While researchers emphasise the resilience of the public sector to changes in organisational culture [23, 36, 37], the management literature argues that an organisation’s culture can change [38, 39], but such changes generally occur very slowly [40]. The organisational culture is considered intricately related to its leadership, particularly its upper-echelon leaders [41, 42]. They are believed to be the primary influence on the creation and development of organisational culture [18], on the beliefs, attitudes, and behaviour of organisational members [43], and they are the managers of culture change [8, 44]. I make this assumption in my study about mayors and their ability to influence the organisational culture of the local administration they manage. In the Polish context, mayors are directly elected political leaders and, at the same time, managers of municipal offices, supervisors of local administration employees. Combining these two functions means integrating political place leadership with organisational leadership. In both place leadership [45] and organisational leadership [46], local leaders are expected to have a more decentralised, adaptive and networked approach.

Directly elected mayors are political leaders selected directly by citizens and head multifunctional local government authorities [47]. Directly electing a mayor means that the citizens of a municipality decide which candidate will take office. In such a process of electing a local government, the individual qualities of the mayors and their personality, as well as the voters’ belief in the candidates’ ability to perform their job competently, are of great importance. Researchers most often take education and professional prestige [48] and motivation for public service and managerial competence [48, 49] as measures of the quality of local political leaders. Carnes and Lupu [50] argue that education and prior occupation of candidates to be political leaders do not matter for governance performance. In contrast, the partisanship of the mayors [51] and their business experience [52] are indicated as factors influencing the way fiscal policy is shaped in municipalities. However, recent research indicates that mayors’ managerial competence determines the success of municipal governance, as measured by population change [48]. Previous research has also confirmed a positive relationship between the seniority in public administration and re-election of local politicians and motivation to serve in public office [53]. Bartnicki proves that mayor multi-tenure strengthens the effectiveness of mayor’s representation of voters’ interests through political experience [54].

However, there is little empirical study of the linkages between leaders’ individual differences and organisational characteristics [55, 56]. The study of Giberson [55] provides initial empirical evidence that organisational culture values are, at least to some extent, a reflection of the CEO’s personality, agreeableness and emotional stability. The literature review reveals a gap in knowledge about the relationship between the characteristics of local public managers and mayors and the organisational culture of the offices they lead. It can be expected that the mayor’s experience in the private sector will support strengthening the characteristics of market culture in the municipality, while the experience in the public sector of hierarchy culture. Indeed, private and public organisations differ in organisational culture due to many characteristics, including different business objectives, values adopted, accountability, control mechanisms, access to resources, and the nature of organisational constraints (economic versus political) [24, 57]. However, the results of comparative studies are not unequivocal. They indicate the greater importance of market culture characteristics in private organisations than in public ones, and hierarchy culture characteristics in public organisations than private ones [23, 58, 59], and vice versa [57, 60]. However, in terms of market culture traits, the corporate sector is clearly distinguished from local government. The mayor’s previous experience of stronger market culture traits may influence his beliefs, attitudes and behaviour, and therefore the organisational culture of the municipality. All more so as values originating in the private sector have gained prominence in the local government through the New Public Management reform. Taking as a starting point the common view of a hierarchical organisational culture in public administration [23, 25], the seniority of mayors in public administration may be relevant to the organisational culture of the municipality.

Mayors as local leaders are predominantly involved in party politics, even if they are not formally attached to any nation-wide party [61]. Political parties nominate political personnel for offices and mandates to exercise power and adopt policies implemented by the public administration [62, 63]. They have an efficient organisation, professionalised party bureaucracy and experts [61]. The question, therefore, arises as to whether the mayor’s membership of a political party reinforces the cultural characteristics of the hierarchy in the municipality.

Mayors have different managerial and political experiences also depending on the term of office they serve. Undoubtedly, one of the important factors for a mayor’s re-election is to perform effectively as a manager, solve local problems, and respond to local challenges, ensuring voter approval. Important for implementing the mayor’s policy agenda is building and maintaining a network of actors on whom the mayor depends in addressing the major issues [64, 65]. On this basis, it can be assumed that with the next re-election, the mayors increase their facilitation and collaborative relationship management skills and awareness of the role of multi-actor networks in local government. This may, in turn, have a bearing on the strength of the clan organisational culture in the municipality. Additionally, the mayor’s previous work experience of being a member of an NGO can be considered a contributing factor to the clan culture in the municipality. This relationship is justified by both the specifics and values of NGO’s, such as solidarity, willingness to help and the findings of researchers [66, 67], that non-governmental organisations (NGO) demonstrated a clan culture.

When analysing the mayor’s previous work experience from the point of view of its relevance to adhocracy culture, it is important to consider the determinants of innovation in the public organisation. According to Hambleton [65], the interest in innovation in public services was initially a response to significant cuts in public spending and the need to seek efficient solutions. However, he found the managerial approach insufficient. The author emphasises that important to the introduction of radical public innovation is usually a process of co-creation—a process in which new solutions are generated by working with people, not for them [65]. Thus, the combination of a mayor’s previous experience from the business sector and NGO’s can shape a set of mayor’s values and competencies that will reinforce the characteristics of an adhocracy culture in the municipality.

Based on all reviews mentioned above (especially researchs’ reviews on the importance of mayors’ managerial, social and political competencies for local policymaking and the influence of organisational leader characteristics on organisational culture) the following hypotheses were formulated:

*H1a*: *There is a positive relationship between hierarchy culture in municipal administration and the seniority of mayors in public administration*, *and the membership of mayors in the political party*.
*H1b*: *There is a positive relationship between market culture in municipal administration and the work experience of mayors in private sectors (employment or self-employment)*.
*H1c*: *There is a positive relationship between clan culture in municipal administration and multi-incumbency of mayors and the experience of mayors in the NGO*.
*H1d*: *There is a positive relationship between adhocracy culture in municipal administration and the hybrid work experience of mayors in NGO’s and private sectors*.

### Leadership style of directly elected mayors and organisational culture of municipalities

Researchers use different classifications of mayor’s leadership styles. For example, John [68], taking into account the distinction between authoritarian and responsive leadership, distinguished the styles: "caretaker", "consensus facilitator", "city boss", "visionary". A similar classification was also developed by Getimis and Hlepas [69]. The four ideal types of leaders were the visionary (cooperative and strategic), the city boss (authoritarian and strategic), the consensus facilitator (cooperative and reproductive) and the protector (authoritarian and reproductive). Getimis and Grigoriadou [70], on the other hand, identified the possible attitudes of leaders towards their role and classified ten leadership styles on this basis: proactive, reactive, competent, consensual, programme politician, caretaker, negotiator, confrontational style, city manager, politician.

Management literature confirms the existence of a relationship between managers’ leadership style and organisational culture. The leader behaviours are more control-oriented in a bureaucratic and supportive culture and more flexible-oriented in innovative culture [71]. Hierarchy culture is negatively connected to relationship-oriented leadership behaviour [72]. On the other hand, Gao [73] showed that leaders with openness to change values form an adhocracy culture, while leaders expressing conservative values promote a hierarchy culture.

Research on the leadership style of mayors in Europe [74] shows that in the 2000s, there was a shift in mayor leadership style from one dominated by an authoritarian attitude to one open and more cooperative. A visionary leadership style became the most common among mayors. The study found that the leadership style of mayors is linked to their professional practices in the public and private sectors. Mayors from the private sector were more cooperative, and mayors from the public were more strategically oriented. Kim and Yoon [75] demonstrated that the cultivation of a culture of innovation in local government relies on senior managers’ transformational leadership and the climate for creativity. Research on the leadership style preferences of Polish mayors concludes that they influence the priorities adopted in local politics [76]. More participatory and solidarity-based management approaches of mayors foster higher prioritise modern policy/practice areas and interventions to tackle new urban sustainability challenges.

Based on the information presented above, the following hypotheses were formulated:

*H2a*: *There is a positive relationship between hierarchy culture in municipal administration and the political-oriented leadership style of mayors*.
*H2b*: *There is a positive relationship between market culture in municipal administration and the managerial-oriented leadership style of mayors*.
*H2c*: *There is a positive relationship between clan culture in municipal administration and the cooperation-oriented and solidarity-oriented leadership style of mayors*.
*H2d*: *There is a positive relationship between adhocracy culture in municipal administration and the mayor’s visionary-oriented and cooperation-oriented leadership style*.

## Materials and methods

### Data collection

The research was carried out by the author at the local government level in Poland in 2018. Data were collected using an anonymous, online survey questionnaire that was administered via Google Forms (S1 Appendix). I used a quantitative approach to study as many municipalities as possible and to be able to make comparisons. The respondents were mayors, who, by the Act on Municipal Self-Government, act at the same time as managers of municipal offices. The mayors possess knowledge regarding the organisation and strategic goals of local governments and, therefore, were considered appropriate respondents to complete the questionnaire. This allowed obtaining an overall picture. The invitations to participate in the study were sent per e-mail to all 2478 Polish municipalities. 1236 mayors took part in the survey (49.9% of the population of municipalities). 319 incorrectly completed OCAI forms were excluded from further analysis. The sample consisted of 917 mayors (37.0% of the population of municipalities) from three municipality types: urban, urban-rural (which include within their administrative boundaries both city/town areas, as well as areas outside city limits), and rural municipalities (S1 Dataset). The data were analysed anonymously, therefore, the author had no access to personal identifying information. Table 1 presents the sample characteristics.

**Table 1:** Sample characteristics.

		urban municipalities (N = 152)		urban-rural municipalities (N = 207)		rural municipalities (N = 558)		all municipalities (N = 917)	
		N	%	N	%	N	%	N	%
**Number of mayoral terms**	1	62	40.8	71	34.3	168	30,1	301	32.8
	2	25	16.4	51	24.6	114	20,4	190	20.7
	3	27	17.8	35	16.9	83	14,8	146	15.8
	4	15	9.9	17	8.2	40	7,2	72	7.9
	above 5	9	5.9	10	4.8	60	10,8	79	8.6
	No answer	14	9.2	23	11.1	93	16,7	130	14.2
**Mayor’s seniority in public administration**	< 5 years	39	25.7	45	21.7	108	19,4	192	20.9
	6–30 years	110	72.4	155	74.9	408	73,1	673	73.4
	> 31 years	3	1.9	7	3.4	42	7,5	52	5.7
**Mayor’s experience of working in the business sector**	yes	44	28.9	78	37.7	166	29.7	288	31.4
	not	51	33.6	77	37.2	223	40.0	351	38.3
	No answer	57	37.5	52	25.1	169	30.3	278	30.3
**Mayor’s experience in running his own business (self-employment)**	yes	27	17.8	26	12.6	86	15.4	139	15.2
	not	66	43.4	110	53.1	291	52.2	467	50.9
	No answer	59	38.8	71	34.3	181	32.4	311	33.9
**Mayor’s political party affiliation**	yes	39	25.7	36	17.4	109	19.5	184	20.1
	not	105	69.1	160	77.3	415	74.4	680	74.2
	No answer	8	5.2	11	5.3	34	6.1	53	5.7
**NGO membership of mayor**	yes	42	27.6	70	33.8	180	32.3	292	31.8
	not	92	60.5	120	58.0	313	56.1	525	57.3
	No answer	18	11.9	17	8.2	65	11.6	100	10.9
**Administration size**	< 30 employees	12	7.9	46	22.2	310	55.8	369	40.2
	31–100 employees	77	50.7	137	66.2	230	41.1	443	48.3
	> 100 employees	57	37.5	20	9.7	0	0.0	77	8.4
	No answer	6	3.9	4	1.9	18	3.1	28	3.1
**Total**		152	100.0	207	100.0	558	100.0	917	100.0

The largest number, over 30% of mayors, did not provide information on having experience of working in the private sector (either as an employee or self-employment). This may be due to mayors’ concerns about negative perceptions of the links between political careers and business.

## Measures

### Organisational culture

The Organisational Culture Assessment Instrument (OCAI) [8] was used to diagnose organisational culture in Polish municipalities. The OCAI uses a four-factor model to classify cultures as falling along two bisecting continua: stability versus flexibility in work approaches and internal versus external focus of the organisation [8]. The OCAI consists of six questions (Dominant Characteristics, Organisational Leadership, Management of Employees, Organisational Glue, Strategic Emphases, Criteria of Success), being core cultural dimensions as perceived by mayors with each question having four alternatives (A = Clan, B = Adhocracy, C = Market, D = Hierarchy). Respondents allocate 100 points among these four alternatives based on how each alternative is similar to the organisation being assessed. More points are allocated to the alternative that is most like the organisation being assessed [8].

In the first step of the analysis, the overall mean and standard deviation for the 24 OCAI items were calculated. This information provides insight into the organisational culture dimensions patterns that predominate across Polish municipalities and within the three Polish municipality types (urban, urban-rural, and rural). Then, the overall average and standard deviation of the number of points scored from the six questions for the four alternatives (types) of organisational culture for all surveyed municipalities were calculated. The higher the score, the stronger and the more dominant a given alternative (type) of organisational culture is in the municipality. The next step of the analysis was to assess the internal reliability of the four alternatives of organisational culture using Cronbach’s alpha statistic. Then, the overall average and standard deviation were calculated for four types of organisational culture in three groups of municipalities separated by type and in three groups of municipalities separated by administration size. This information allowed me to characterise the cultural profiles of municipalities of different types. To estimate the influence of control variables: municipality type and administration size, on the level of particular alternatives (types) of organisational culture, the analyses of variance (ANOVA and UNIANOVA) were used. Levene’s test tested the homogeneity of variance.

### Professional, social and political experience of mayors

Mayors’ experiences were measured using six variables (Table 1):

- seniority in public administration: (1) time working in local government (number of years),
- work experience in the business sector: (2) have experience of working in the business sector (yes/no), (3) have experience in running their own business (yes/no),
- political experience: (4) number of terms as mayor, (5) political party affiliation (yes/no),
- social experience: (6) NGO membership (yes/no).

The next step in my analysis was to test the hypotheses H1a-H1d using ANOVA and UNIANOVA to confirm a relationship between the mayor’s work experience in the public, private, social and political sectors and the organisational culture of the municipal administration. The Tukey HSD multiple comparisons test was used.

### Preferred place leadership style of mayors

The place leadership style of the mayor was perceived as an important factor that influences organisational culture in local administration. I identify five management styles based on the literature [74, 76], namely: (1) a loyal politician who implements the objectives of his political party—political-oriented place leadership; (2) a visionary and strategist who incorporates a long-term perspective into municipal management—visionary-oriented place leadership; (3) an efficient manager who manages the municipality—managerial-oriented place leadership; (4) an initiator/coordinator who shapes relationships, cooperation and networks in city management—cooperation-oriented place leadership; and (5) a leader guided by the common interest and solidarity—solidarity-oriented place leadership. Respondents expressed their preferences for these management styles through a five-point Likert scale (1 = definitely disagree with the specific style, to 5 = definitely agree with the specific style).

In the next step, an analysis of variance ANOVA and UNIANOVA to test hypotheses H2a-H2d were conducted on the relationship between mayor leadership style and organisational culture of the municipal administration.

## Results

### Dominant culture type in Polish municipalities

The diagnosis of the cultural profiles of the surveyed municipalities using the OCAI confirmed that their dimensions show characteristics of mainly two types of organisational cultures: hierarchy and clan (Table 2). The hierarchy culture and the associated emphasis on policies, rules and stability characterise 2 of the 6 dimensions: organisational leadership and criteria of success. Organisational leadership examined municipalities focus on coordination and efficiency-mindedness. Criteria for success, on the other hand, are dependability, efficiency, and stability. The characteristics of clan culture and its typical emphasis on a common vision, collaboration, and employee commitment, occur in 3 of the 6 dimensions: management of employees, the organisational glue and strategic emphases. They focus on the same cultural values. Management of employees focuses on consensus, cooperation, and teamwork. The organisational glue is cooperation, mutual trust and loyalty. Moreover, strategic emphases focus on a common vision and collaboration. Market culture, characterised by results-oriented and attainment of determinable goals and targets, defines only one dimension—"dominant characteristic". The adhocracy culture was not dominant in any of the dimensions. It can be concluded that the organisational culture does not demonstrate total congruence. This indicates either “the culture is unclear to respondents or the complexity of the environment requires multiple emphases in different areas of the organisation” [8]. The incongruence of culture is often a sign of the need for cultural change.

**Table 2:** Dominant culture type by dimensions in polish municipalities.

Dimensions	Dominant Culture type	M	SD	Dominant Culture type	M	SD	Dominant Culture type	M	SD	Dominant Culture type	M	SD
	Total (N = 917)			Urban municipalities (= 152)			Urban-rural municipalities (N = 207)			Rural municipalities (N = 558)		
**Dominant characteristic**	Market	34	17.14	Market	34	17.38	Market	34	16.55	Market	34	17.60
**Organisational leadership**	Hierarchy	40	20.04	Hierarchy	40	20.02	Hierarchy	40	20.34	Hierarchy	40	20.18
**Management of employees**	Clan	42	21.46	Clan	42	21.40	Clan	42	22.27	Clan	42	21.12
**Organisational glue**	Clan	36	18.64	Clan	36	18.53	Clan	34	17.95	Clan	38	19.03
**Strategic emphases**	Clan	33	17.12	Clan	33	17.01	Clan	32	17.33	Clan	34	17.22
**Criteria for success**	Hierarchy	37	19.08	Hierarchy	37	18.50	Hierarchy	37	18.40	Hierarchy	36	19.52

The highest mean score was the management of employees (M = 42, SD = 21.46—total municipalities; M = 42, SD = 21.40—urban municipalities; M = 42, SD = 22.27—urban-rural municipalities; M = 42, SD = 21.12—rural municipalities), with clan dominant culture type. The dominant organisational culture in individual dimensions remains the same in municipalities of different types. The high standard deviation for the individual culture dimensions indicates a large variation in how the mayors perceive them. The high values are because each respondent assessed the organisational culture of the municipality they manage, and the standard deviation was calculated based on the assessments of all respondents representing 917 municipalities.

Table 3 shows the average results of all six dimensions’ means by cultural alternatives. The dominant organisational culture in municipalities is the clan culture, whose most prominent traits are human resources and employee commitment. However, it is worth noting that a second strong archetype is the hierarchy culture, which focuses on efficiency, stability, and dependability. Comparing these results with Public Administration reference culture (average culture profile for Public Administration: clan (M = 21), adhocracy (M = 13), hierarchy (M = 32), market (M = 23)), obtained from the observations of Cameron and Quinn [8], it can be concluded that Polish municipal offices are more focused on consensus, cooperation, and teamwork than reference offices. The municipalities surveyed are neither innovators nor organisations with entrepreneurial organisational culture, but the average scores for adhocracy culture are significantly higher, compared to the Public Administration reference culture. The Cronbach’s alpha coefficients (α-reliability statistic) ranged from 0.65 to 0.76, so the participants’ answers were considered reliable. The dominant organisational culture in rural municipalities is clan culture—unlike in other urban municipalities, where hierarchy culture dominates. On the other hand, considering the level of employment in administration, the dominant type of organisational culture in each group of municipalities is clan culture.

**Table 3:** The profile of organisational culture in municipal offices in Poland.

		Clan	Adhocracy	Market	Hierarchy
**Total municipalities**	M	**31.11868411**	20.71156	18.01708	**30.15085**
	SD	11.16658247	8.290941	7.77837	11.69276
	α Reliability	0.7	0.76	0.65	0.72
**Type of municipality (N = 917)**					
**Urban municipalities (N = 152)**	M	26.55834	22.20519	19.61834	**31.32514**
	SD	10.01433	8.4442	8.095067	12.78296
**Urban-rural municipalities (N = 207)**	M	30.00796	21.67525	18.79543	**30.82931**
	SD	11.45484	8.698604	7.633552	12.24208
**Rural municipalities (N = 558)**	M	**32.82133**	17.82787	14.25798	29.58781
	SD	11.25293	8.045591	7.795625	11.28851
**Administration size (N = 889)**					
**<30 employees (N = 369)**	M	**31.1187**	20.7116	18.0171	30.1509
	SD	11.1666	8.2909	7.7784	11.6928
**31–100 employees (N = 443)**	M	**31.0175**	20.7716	18.0552	30.1538
	SD	11.0889	8.2465	7.7909	11.7073
**>101 employees (N = 77)**	M	**30.9956**	20.7421	18.0420	30.2185
	SD	11.0908	8.2569	7.7891	11.7108

To examine the effect of municipality type and administration size on the level of each type of organisational culture (dependent variable), ANOVA and UNIANOVA analyses of variance were conducted (Table 4). They confirmed the statistically significant effect of municipality type on the level of market (F(2, 880) = 6.605; p = 0.002; η2 = 0.014), clan (F(2, 880) = 4.338; p = 0.013; η2 = 0.010) and adhocracy organisational culture type in municipalities (F(2, 880) = 3.683; p = 0.026; η2 = 0.008), and in the case of market culture type there was also the interaction between municipality type and administration size (F(4, 880) = 2.537; p = 0.039; η2 = 0.011). However, this effect is weak (municipality type explains 1.4% of market culture type variation (η2 = 0.014), 1% of clan culture variation (η2 = 0.010), less than 1% of adhocracy culture variation (η2 = 0.008). The type of municipality also differentiates the level of market culture by administration size to a small extent (explaining about 1.1% of the variation in market culture type - η2 = 0.011). The analysis did not confirm the effect of municipality type and administration size on hierarchy culture type (they do not statistically significantly differentiate the hierarchy culture type variable). It was not possible to use a two-factor analysis of variance to test the influence of both factors due to failure to meet the assumption of homogeneity of variance across subpopulations.

**Table 4:** Results of analysis of variance: Municipality type, administration size and organisational culture types.

	Hierarchy culture			Market culture				Clan culture				Adhocracy culture			
	F	df	p	F	df	p	η2	F	df	p	η2	F	df	p	η2
**Type of municipality**	1.613	2	.200	6.605	2	.002*	0,014	4.338	2	.013*	0,010	3.683	2	.026*	0,008
**Administration size**	.057	2	.994	1.393	2	.249	0,03	.706	2	.494	0,002	.824	2	.439	0,002
**Type of municipality and employment level**	-	-	-	2.537	4	.039*	0,011	.497	4	.738	0,002	1.975	4	.096	0,09

Note

*p> 0.05.

Based on the results of the paired comparisons test for the variable type of municipality, it can be indicated that statistically significant differences exist between rural (M = 14.275, SD = 1.312) and urban-rural municipalities (M = 18.795, SD = 0.727) and urban municipalities (M = 19.618, SD = 0.872) in terms of market culture. On average, the rural municipality will have a lower level of market culture than the representatives of the other two groups of municipalities. For clan culture, statistically significant differences exist between rural (M = 32.821, SD = 1.891) and urban (M = 26.558, SD = 1.257) municipalities. On average, a rural municipality will have a higher level of clan organisational culture than an urban municipality. For adhocracy culture, on the other hand, statistically significant differences occur between a rural municipality (M = 17.827, SD = 1.396) and urban-rural municipality (M = 21.675, SD = 0.774) and urban municipality (M = 22.205, SD = 0.928). On average, the rural municipality will be characterised by a lower level of adhocracy organisational culture than the representatives of the other two groups of municipalities. Thus, there are associations between the level of urbanisation of municipalities and the strength of clan, adhocracy and market culture.

### Work experience of mayors and organisational culture type

I analysed the work experience of mayors from several points of view: political experience (number of terms of the mayor and political party affiliation) and work in public, private and social sectors. This data is presented in Table 1. 32.8% of mayors have been in the office for the first time, 20.7% for the second time and almost 9% for more than four times (i.e. more than 16 years). 48.3% of the mayors manage offices with 30 to 100 employees, while 40% manage small organisations with up to 30 employees. Large offices with more than 100 employees are managed by more than 8% of those surveyed. Only 20% of the mayors are members, and 74.2% are not members of a political party, while the rest did not disclose such information. The majority of mayors (94%) have worked in local administration, and 67.6% of them have long-term experience of more than six years as an official. Far fewer mayors have work experience in the private sector. More than 30% of the mayors were employed in the private sector and about 15% were self-employed. Less than 32% of the mayors were members of an NGO.

To test the hypotheses H1a-H1d concerning the relationship between the political and professional experience of the mayor and the strength of organisational cultures, I conducted analyses of variance ANOVA (with one factor differentiating the mean) and UNIANOVA (with more than one factor differentiating the mean). The results are presented in Table 5.

**Table 5:** Results of analysis of variance for testing hypotheses H1a-H1d.

Models	F	df	p	η2
**H1a**	**Hierarchy culture**			
**Political party affiliation**	.849	1	. 357	0,001
**Seniority in local public administration**	3.040	2	.048*	0,007
**Political party affiliation and seniority in local public administration**	0.294	2	. 746	0,001
**H1b**	**Market culture**			
**Professional experience in the business sector**	.715	1	.398	0,001
**Experience in running your own business**	.063	1	.802	0,000
**Professional experience in the business sector and in running your own business**	.088	1	.767	0,000
**H1c**	**Clan culture**			
**NGO membership**	5.433	1	.020*	0,008
**Number of mayoral terms**	1.354	4	.248	0,008
**NGO membership and number of mayoral terms**	1.210	4	.305	0,007
**H1d**	**Adhocracy culture**			
**Work experience in the private sector (employment or self-employment)**	2.089	1	.149	<0,003
**NGO affiliation**	0.192	1	. 661	<0,000
**Work experience in the private sector (employment or self-employment) and NGO affiliation**	0.868	1	.352	0,001

Note

*p> 0.05.

The analysis of variance for H1a identified the mayor’s work experience in local public administration as a factor that statistically significantly affects the level of the dependent variable–the hierarchy culture type (F(1, 816) = 3.040; p = 0.048; η2 = 0.007). The F-test probabilities for party affiliation and the interaction effect of the two variables exceed the significance level of p>0.05, which does not allow me to identify them as statistically significant. Hence, hypothesis H1a was positively verified only about seniority. The length of the mayor’s seniority in public administration differentiates the degree of hierarchization of the organisational culture of the office while keeping the mayor’s party affiliation status unchanged. However, the effect of this factor is weak (the factor level explains less than 1% of the variation in culture type).

Based on the results of the multiple comparisons test (Tukey HSD test) for the variable seniority in public administration, it can be indicated that statistically significant differences occur between groups of mayors with seniority up to 5 years (M = 32,551, SD = 1,102) and a group of 6–30 years (M = 29,736, SD = 0,574). On average, the municipal office headed by a mayor with less than 5 years of seniority will be characterized by a higher level of hierarchization of organisational culture than the office headed by a mayor with between 6 and 30 years of seniority.

The analysis of variance for H1b did not identify factors that would statistically significantly affect the level of the market culture variable. The probabilities in the F-test for both variables (the mayor’s experience in working in the business sector and in running his own business) and the interaction effect between them exceed the significance level, p>0.05. There is no basis for considering the hypothesis posed as true based on the survey results obtained.

The analysis of variance for H1c identified the mayor’s NGO membership as a factor that significantly affects the level of the dependent variable—clan culture type (F(1, 712) = 5.433; p = 0.020; η2 = 0.008). The probabilities in the F-test for the second variable considered in the hypothesis—the number of mayor’s terms of office and the interaction effect between the two variables exceeds the significance level of p>0.05. H1c was therefore partially positively verified only about the mayor’s NGO membership. With the constant number of terms of office of the mayor, his NGO membership increases the mean level of the variable type of culture (M = 33.506, SD = 0.802) compared to a situation in which the mayor does not belong to this type of organisation (M = 30.506, SD = 0.632). However, the effect of this factor is weak (explains less than 1% of the variation in culture type).

The analysis of variance for H1d did not identify private sector work experience and NGO affiliation as factors that would statistically significantly affect the level of the dependent variable. The F-test probabilities for both variables and the interaction effect exceed the significance level, p>0.05 (NGO affiliation—F(1, 813) = 0.192, p = 0.661, η2<0^.^000; private sector experience—F(1, 813) = 2.089, p = 0.149, η2<0.003; combined effect of both factors—F(1, 813) = 0.868, p = 0.352, η2 = 0.001). There are no grounds to consider the hypothesis put forward as being supported by the results of the study.

### Preferred place leadership style of mayors and organisational culture type

The preferred place leadership style of the mayors was identified based on respondents’ ratings for five types of leadership styles (Table 6). The respondents used the Likert scale from 1 to 5 (1 = definitely disagree with the specific style, to 5 = definitely agree with the specific style). 4 out of 5 analysed styles (visionary-, managerial-, cooperation- and solidarity-oriented) were rated similarly by the vast majority of mayors, who gave them high ratings of 4 and 5. On the other hand, the agile politician style received the lowest ratings.

**Table 6:** Preferred place leadership style of mayors.

Leadership style	1	2	3	4	5	M	Me
**A Loyal Politician who Implements the Objectives of His/Her Political Party—political-oriented place leadership**	236	454	158	46	11	3.21	2
**A Visionary and Strategist who Incorporates A Long-Term Perspective into Municipal Management–visionary-oriented place leadership**	3	18	50	443	396	4.33	4
**An Efficient Manager who Manages the Municipality—managerial-oriented place leadership**	4	5	31	438	430	4.42	4
**An Initiator/ Coordinator who Shapes Relationships, Cooperation and Networks in City Management—cooperation-oriented place leadership**	2	12	58	501	334	4.27	4
**A Leader Guided by the Common Interest and Solidarity—solidarity-oriented place leadership**	3	2	40	471	391	4.37	4

To test the H2a-H2d hypotheses were conducted ANOVA analyses of variance for one factor differentiating the mean and UNIANOVA for more than one factor differentiating the mean. The results are presented in Table 7. The analysis did not identify factors that would significantly affect the level of the dependent variable in each hypothesis tested. The probability in the F-test for each analysed factor for each tested hypothesis exceeds the adopted significance level of p>0.05, which means that the place leadership style of mayors assumed in the hypotheses does not affect the assumed higher value of the organisational culture type. The hypotheses H2a-H2d were rejected.

**Table 7:** Results of analysis of variance for testing the H2a-H2d hypotheses.

Models	F	df	p
**H2a**	**Hierarchy culture**		
**Political-oriented place leadership**	1.591	4	.175
**H2b**	**Market culture**		
**Managerial-oriented place leadership**	.816	4	.515
**H2c**	**Clan culture**		
**Cooperation-oriented and solidarity-oriented place leadership**	2.162	4	.071
**H2d**	**Adhocracy culture**		
**Visionary-oriented place leadership**	.859	4	.488
**Cooperation-oriented place leadership**	1.531	4	.191
**Visionary-oriented and cooperation-oriented place leadership**	.779	8	.622

## Discussion

The analyses indicate that the dominant organisational culture in the offices of Polish municipalities is the clan culture. The second in order is the hierarchy culture. The conclusion about the co-dominance of the clan and hierarchy culture in Polish municipalities, especially urban-rural municipalities, is consistent with the findings of other authors [3, 27, 28].

Strong features of clan culture are demonstrated in Polish municipalities by the following dimensions: management of employees, organisational glue, and strategic emphases. Co-operation, consensus, and loyalty are factors on which, according to the mayors, the relations between local administration employees are based. Cooperation is also a factor in building organisational cohesion in municipalities’ offices, which is why development is emphasised. This is an essential finding, as it assumes that self-government employees are open to sharing their knowledge, complementing skills, striving to work out compromise decisions. This should be conducive to the effective implementation of public tasks.

The strong manifestation of clan characteristics in the organisational culture of Polish municipalities may result from the specificity of the local government administration and the conditions of its operation. First of all, it pursues common public goals, the mission of sustainable development and builds the sustainable communities. These tasks are characterised by high complexity, resulting from the need to integrate actions for social and spatial cohesion, local economic development, ecology and resource efficiency, or the development of urban intelligence. The diversity of problems and possible solutions and the requirement for transparency and fairness in the use of resources make that these goals require an integrated and comprehensive approach of local government managers to the local development policy and building strong relations with inhabitants and other stakeholders [76]. Municipalities are obliged by law to work with residents and NGOs through participatory processes and are involved in numerous inter-municipal cooperation projects and cross-sectoral partnerships [77]. It is the officials who are directly responsible for initiating, coordinating and implementing cooperation projects with stakeholders. Another factor influencing strong clan characteristics in municipalities may be the unstable and chaotically changed law [78], based on which Polish municipalities take actions. Changes in the law are not comprehensive and consistent but piecemeal and frequent, which causes difficulties in interpreting and complying with the law. This phenomenon creates uncertainty and may induce officials to cooperate internally and cooperate with government offices to avoid mistakes in the interpretation of regulations. Clan culture is also fostered by managers’ long professional experience in public administration, as already pointed out by other authors [27, 33].

The results of my research indicate that clan culture is stronger in rural municipalities than in urban municipalities. This finding also stems from other Polish studies [20]. The offices of rural municipalities are small organisations where cooperative relationships are more easily formed. Moreover, the distance between the authority and administration and the inhabitants in rural municipalities is smaller and is based on close relations.

The second strong organisational culture in Polish municipalities is the culture of hierarchy. It is stronger than clan culture in urban municipalities. In particular, it dominates in the areas of organisational leadership and criteria of success. Organisational leadership in the hierarchy culture is identified with coordinating, efficiently organising, creating harmonious conditions for achieving good results. On the other hand, cost-effectiveness is taken as a criterion of success. Maintaining liquidity, meeting schedules and low service costs are the most important.

The great importance of the features of hierarchical culture in the cultural profiles of municipalities undoubtedly results from the principle of legalism and formalisation of local government activities. Actions taken by municipalities must have a legal basis, and legal acts taken by municipalities are controlled for legality by representatives of the government administration in voivodeships (regional self-government communities, the highest level of territorial division in Poland)—the voivodes, and in the financial sphere also by Regional Chambers of Audit. According to a recent comparative study of the local autonomy index [79], Polish municipalities are among the most autonomous local governments in Europe, after Switzerland and Scandinavian countries. Nevertheless, a recentralisation trend has been observed in recent years [78]. What can be seen is a self-government gradual deprivation of control over subsequent areas of tasks and a tightening of supervision over self-government [80], which leads to an increasing number of government administration guidelines and an increase in formalisation. The hierarchy culture may also be strengthened by the procedures of spending and accounting for EU subsidies by municipalities. This is because the inflow of money in Poland is connected with the implementation of specific management methods, patterns of legal regulations, and patterns of organisational structures. Analyses by J. Hryniewicz [81] indicate that the intensive cultural import as a result of the implementation of the EU cohesion policy in Poland has contributed to a large increase in the bureaucratic efficiency of the regional public administration but has not resulted in a clear transformation of the organisational culture towards a market one. It is worth adding that local government units in Poland are significant beneficiaries of EU funds. Local governments have obtained in 2014–2020 more than 60 bn. subsidies, which is about 31% of the EU funding for Poland [82], and thanks to these subsidies, local governments have financed more than 40% of their investment expenses in 2014–2020 [83]. The pressure to obtain and correctly account for grants is reinforced by the difficult financial situation of local governments.

The obtained results indicate the low importance of market culture and adhocracy characteristics in the cultural profile of municipalities’ offices. Market culture dominates only in the “dominant characteristic” dimension, which means that municipalities’ focus on results and the best possible performance of tasks is strong. The following factors may contribute to this: municipalities’ pursuit of the objectives laid down in the development strategies, pressure on meticulous accounting for the disbursed EU funds, annual reporting of results (mayors are obliged to prepare a report on the state of the municipality, which is subject to the vote of the municipal council and the basis for granting the mayor’s vote) as well as mayors’ striving for high positions in local government rankings to promote their municipality or to build individual electoral capital. Such rankings are published annually by numerous organisations, e.g., the local government magazine “Wspólnota”, the newspaper “Rzeczpospolita”, local government associations and others. Moreover, market characteristics are more strongly manifested in municipalities with a higher level of urbanisation. In turn, the culture of adhocracy is not dominant in any area. It increases with the level of urbanisation of the municipality and is most strongly manifested in cities. The researchers indicate that adhocracy culture fosters innovation in organisations [84–86]. It is not surprising that the Polish cities are the municipalities with the highest level of innovation [87].

This uneven distribution of cultural archetypes, the dominance of clan and hierarchical characteristics over market and adhocracy characteristics, may result in a relatively good ability of municipalities to realise the postulate of hybrid governance, especially networked, stakeholder governance, but lower in terms of market-driven governance and experimental governance and consequently lower innovation of municipalities. Strong characteristics of clan culture can foster collaborative processes with stakeholders, and rule abidance can be seen as an organisational good rather than an organisational hindrance [88]. On the other hand, previous analyses indicate the difficulties of Polish municipalities in forming especially partnership relations in the economy [89] and with the inhabitants [90]. Moreover, other authors have shown that strong features of hierarchical culture negatively influence public managers’ attitudes towards cooperation with residents [91]. Both the low level of adhocracy features in organisational culture and the signaling problems of Polish municipalities in implementing cooperation with entrepreneurs and inhabitants may indicate the low competence of municipalities in the field of experimental governance.

The analyses showed a statistically significant relationship between mayors’ work experience in the public and social sector and the strength of particular cultural archetypes in the cultural profile. Firstly, it was confirmed that the seniority of the mayor in public administration has a (weak) influence on the strength of the hierarchy culture. It is higher for mayors with shorter seniority. This can be explained by the mayor’s greater emphasis as an office manager on procedures at the beginning of the political leader’s career due to insufficient knowledge of the specifics of the local administration’s operations and a strong reliance on procedures to avoid mistakes.

My research also confirmed a statistically significant effect of the mayor’s membership in an NGO on the strength of the clan culture. This means that previous experience in the NGO sector, which operates based on the values of solidarity, cooperation, and common good, is conducive (albeit to a small extent) to building a climate for cooperation in the office. This conclusion is consistent with the opinions of other authors regarding the importance of the previous experience of managers in the non-governmental sector for the inclination to social participation [91]. It is worth noting that increasing candidates representing local associations and committees take part in local elections in Poland. It can be assumed that a greater representation of community activists in the ranks of mayors will foster a greater prevalence of clan characteristics over bureaucratic ones in the cultural profiles of municipalities.

The multi-incumbency of the mayor, although it strengthens the effectiveness of the mayor’s representation of the voters’ interests thanks to his political experience [54], does not, in the light of the results of my research, strengthen the clan-like features of the organisational culture of Polish municipalities. Thus, the conclusion of other researchers that the long tenure of public managers leads to a strengthening of relations with residents and fosters the building of cooperative relations has not been confirmed [92]. It is worth noting that the multi-incumbency of mayors is a common phenomenon in Poland [93], especially in rural municipalities [54]. The lack of relationship with the strength of the clan culture may be explained by the fact that in many rural municipalities, multi-incumbency is a result of the lack of counter-candidates in elections or low electoral competition, rather than the effective implementation of the role of manager and administrator of local affairs and cooperation with local stakeholders [54]. Furthermore, re-election may be due to the effects of informal ties in the local environment, which may construct and maintain local clientelistic arrangements [54]. However, this is difficult to verify.

The study also did not confirm the relevance of the work experience of private sector mayors to the strength of market culture characteristics. Martin and Kloot [27] demonstrated that hiring managers in local government from the market-oriented business sector did not change the organisational culture in the Australian local government. Most likely, some of those who came from the private sector appear to have moved on, possibly unable to effect the culture change needed to entrench New Public Management and contemporary managerial technologies.

The political experience resulting from membership in a political party does not reinforce the hierarchical characteristics of organisational culture. Polish mayors declare to a large extent to be non-partisan (74% of mayors in the sample and, according to other studies, 60% of Polish mayors [94]). The weakening of the importance of political parties in European local government results from the introduction of direct mayoral elections [95].

In summary, the mayor’s previous work experience in the public and social sectors can potentially influence the cultural profile, reinforcing his clan characteristics. In contrast, previous political and business experience does not reinforce the hierarchy and market culture.

Previous research has confirmed the correlation between strategic and visionary management of municipalities and collaborative organisational culture [96]. The analysis of the relationship between the place leadership style of Polish mayors and the type of organisational culture conducted in this article was the basis for rejecting all hypotheses assumed on this topic. Lack of relationship can be explained by the relatively even distribution of preferences by mayors for 4 out of 5 analyzed leadership styles. Thus, Polish mayors respond to the diverse challenges of community management with a hybrid leadership style. Similar findings emerge from analyses of the identity of Austrian officials and the forms and practices they use in response to competing institutional logics (bureaucracy and New Public Management) [97].

The low degree of explanation by the analysed predictors or their complete lack of significance for the diversity of cultural profiles of Polish municipalities indicate the difficulties of cultural change initiated only at the level of the top management of the office. Such a change, to be effective, should be planned and accepted by all employees. The results, therefore, point to the existence of other predictors. Some previous studies on the importance of demographic variables indicate that organisational culture is partly determined by gender [98, 99]. Due to the high employment rate of women in local administration (65%) [100], it can be assumed that this will be a significant predictor explaining the differences in cultural profiles of Polish municipalities. This relationship requires detailed research.

The results of this study are subject to the usual limitations of on-line surveys, including biases that can arise in respondents’ ratings based on their perceptions or the dishonesty of participants with survey responses. The organisational culture assessment was conducted among mayors, who are the managers of municipalities in Poland, thus representing only their point of view and perceptions and not those of the whole management team. Having a single respondent represent each municipality instead of multiple respondents could be a limitation if the response did not represent the municipality’s culture.

## Conclusion

The research results presented in this article allow for a better understanding of the characteristics of the local government administration. Organisational culture in Polish municipalities is dominated by clan and hierarchy features, and only to a small extent does it manifest adhocracy and market features. This constellation indicates relatively favourable conditions for initiating and carrying out cooperation projects with stakeholders but is insufficient for experimenting and generating innovations. The hierarchical features of local administration are still strong, which confirms partly the limited ability of public managers to influence cultural change and the resilience of the traditional organisational culture model in Polish municipalities. However, the results presented in this article also indicate the diversity of organisational culture in municipalities of different types and in municipalities that mayors manage with specific professional experience. In rural municipalities and municipalities managed by mayors with previous professional experience in the social sector, clan characteristics of organisational culture are manifested more strongly than in the others. On the other hand, the culture of urban municipalities is more strongly characterized by innovation and flexibility.

Changing the cultural profile of the municipality by strengthening clan characteristics can therefore be initiated by having a candidate with experience in the NGO sector as mayor. However, regarding strengthening adhocracy culture, a change in the mayor’s office is not enough. Therefore, a planned change process for the organisational culture and training on cultural change management in municipalities is necessary. These research results also have implications for the initiation and implementation of innovative projects by municipalities. Urban municipalities have potentially the best cultural conditions for cooperation projects and urban experimentation. A cultural change that reinforces the characteristics of adhocracy culture can increase municipalities’ capacity to initiate and implement urban experiments. These findings also point to the need for further research on the demographic predictors of organisational culture in local government and the influence of the cultural profiles of municipal offices on the initiation and execution of cooperation projects and local experiments.

## Supporting information

S1 Appendix Questionnaire (in English and Polish). (DOC)

S1 Dataset Raw data from the questionnaire. (XLSX)
