# Supplementary material for: The relationship between the professional, social, and political experience and leadership style of mayors and organisational culture in local government. Empirical evidence from Poland
Source: PLoS One. 2021 Dec 1;16(12):e0260647. doi: 10.1371/journal.pone.0260647 (PMC8635353; doi:10.1371/journal.pone.0260647)
Supplement: S1 Appendix — (DOC) [file pone.0260647.s001.doc]

# Questionnaire (English version)

Part 1 Organisational culture of municipalities

1.1. PLEASE CHARACTERISE THE ORGANISATIONAL CULTURE OF YOUR OFFICE BY DIVIDING 100 POINTS, AT YOUR DISCRETION, BETWEEN THE 4 STATEMENTS BELOW. THE MORE YOU AGREE WITH A STATEMENT, THE MORE POINTS YOU SHOULD ASSIGN TO IT. THE MORE YOU AGREE WITH A STATEMENT, THE MORE POINTS YOU SHOULD GIVE IT. THE ANSWER THAT COMES CLOSEST TO THE SITUATION IN YOUR OFFICE SHOULD RECEIVE THE MOST POINTS. Please use scores of 0, 5, 10, 15, 20, 25, ...., 90, 95, 100). The points awarded must add up to 100.

| The municipality’s office is a very personal place. It is like a extended family. People seem to share a lot of themselves. |  |
| --- | --- |
| The municipality’s office is a very dynamic and entrepreneurial place. People are willing to take risks. |  |
| The municipality’s office is very results oriented. A major concern is with getting the job done. People are very competitive and achievement oriented. |  |
| The municipality’s office is very controlled and structured place. Formal procedures generally govern what people do. |  |
| TOTAL | 100 |

1.2. PLEASE CHARACTERISE THE LEADERSHIP IN YOUR OFFICE BY ALLOCATING 100 POINTS TO THE 4 STATEMENTS BELOW AS YOU SEE FIT. THE MORE YOU AGREE WITH A STATEMENT, THE MORE POINTS YOU SHOULD GIVE IT. THE MORE POINTS YOU AGREE WITH A STATEMENT, THE MORE POINTS YOU SHOULD GIVE TO IT. Please use scores of 0, 5, 10, 15, 20, 25, ...., 90, 95, 100). The points awarded must add up to 100.

| Leadership in the municipality’s office is generally considered to exemplify mentoring, facilitating or nurturing. |  |
| --- | --- |
| Leadership in the municipality’s office is generally considered to exemplify entrepreneurship, innovation or risk taking. |  |
| Leadership in the municipality’s office is generally considered to exemplify decisive, demanding and result oriented focus. |  |
| Leadership in the municipality’s office is generally considered to exemplify coordinating, organising or efficiency. |  |
| TOTAL | 100 |

1.3. PLEASE CHARACTERISE MANAGEMENT STYLE IN YOUR OFFICE BY DIVIDING 100 POINTS BETWEEN THE 4 STATEMENTS BELOW. THE MORE YOU AGREE WITH A STATEMENT, THE MORE POINTS YOU SHOULD GIVE IT. THE MORE POINTS YOU AGREE WITH A STATEMENT, THE MORE POINTS YOU SHOULD GIVE TO IT. Please use scores of 0, 5, 10, 15, 20, 25, ...., 90, 95, 100). The points awarded must add up to 100.

| The management style in the municipality’s office is characterised by teamwork, consensus and participation. |  |
| --- | --- |
| The management style in the municipality’s office is characterised by individual risk taking, innovation, freedom and uniqueness. |  |
| The management style in the municipality’s office is characterised by hard-driving competitiveness, high demands and achievement. |  |
| The management style in the municipality’s office is characterised by security of employment, conformity, predictability and stability in relationship. |  |
| TOTAL | 100 |

1.4. PLEASE CHARACTERISE THE CONSISTENCY OF YOUR OFFICE BY DIVIDING 100 POINTS BETWEEN THE 4 STATEMENTS BELOW AS YOU SEE FIT. THE MORE YOU AGREE WITH A STATEMENT, THE MORE POINTS YOU SHOULD GIVE IT. THE MORE POINTS YOU AGREE WITH A STATEMENT, THE MORE POINTS YOU SHOULD GIVE TO IT. THE ANSWER WHICH COMES CLOSEST TO THE SITUATION IN YOUR OFFICE SHOULD BE GIVEN THE MOST POINTS. Please use scores of 0, 5, 10, 15, 20, 25, ...., 90, 95, 100). The points awarded must add up to 100.

| The glue that holds the municipality’s office together is loyalty and mutual trust. Commitment to the authority runs high. |  |
| --- | --- |
| The glue that holds the municipality’s office together is commitment to innovation and development. There is an emphasis being on the cutting edge. |  |
| The glue that holds the municipality’s office together is the emphasis achievement and goal accomplishment. Expansiveness and a desire to win are common motivators. |  |
| The glue that holds the municipality’s office together is formal rules and policies. Maintaining a smooth-running organisation is important. |  |
| TOTAL | 100 |

1.5. PLEASE CHARACTERISE THE MANAGEMENT RULE IN YOUR OFFICE BY DIVIDING 100 POINTS BETWEEN THE 4 STATEMENTS BELOW AS YOU SEE FIT. THE MORE YOU AGREE WITH A STATEMENT, THE MORE POINTS YOU SHOULD GIVE IT. THE MORE POINTS YOU AGREE WITH A STATEMENT, THE MORE POINTS YOU SHOULD GIVE TO IT. Please use scores of 0, 5, 10, 15, 20, 25, ...., 90, 95, 100). The points awarded must add up to 100.

| The municipality’s office emphasises human development. High trust, openness and participation persist. |  |
| --- | --- |
| The municipality’s office emphasises acquiring new resources and creating new challenges. Trying new things and prospecting for opportunities are valued. |  |
| The municipality’s office emphasises competitive actions and achievement. Achieving ambitious goals and winning is important. |  |
| The municipality’s office emphasises permanence and stability. Efficiency, control and smooth operations are important. |  |
| TOTAL | 100 |

1.6. PLEASE CHARACTERISE THE MEASURE OF SUCCESS IN YOUR OFFICE BY DIVIDING 100 POINTS BETWEEN THE 4 STATEMENTS BELOW AS YOU SEE FIT. THE MORE YOU AGREE WITH A STATEMENT, THE MORE POINTS YOU SHOULD GIVE IT. THE MORE YOU AGREE WITH A STATEMENT, THE MORE POINTS YOU SHOULD GIVE IT. Please use scores of 0, 5, 10, 15, 20, 25, ...., 90, 95, 100). The points awarded must add up to 100.

|  | The municipality’s office defines success on the basis of human resources, teamwork, employee commitment and concern for people. |
| --- | --- |
|  | The municipality’s office defines success on the basis of having the most original and innovative solutions and becoming an innovation leader. |
|  | The municipality’s office defines success on the basis of winning in the marketplace and occupation the competition. Achieving leadership in the office is most important. |
|  | The municipality’s office defines success on the basis of efficiency. Reliability of public service delivery, smooth scheduling and low costs are critical. |
| 100 | TOTAL |

Part 2 Preferred leadership style of the mayor

2. DO YOU AGREE OR DISAGREE WITH THE FOLLOWING STATEMENTS?

|  | Statements | Strongly disagree -1 | Disagree - 2 | Neither disagree nor agree -3 | Agree - 4 | Strongly agree - 5 |
| --- | --- | --- | --- | --- | --- | --- |
| 2.1. | A mayor is first and foremost an efficient politician implementing the programme of his/her party |  |  |  |  |  |
| 2.2. | A mayor is, above all, a visionary and a strategist, taking into account the long-term perspective in managing a municipality |  |  |  |  |  |
| 2.3. | A mayor is, above all, an efficient manager who manages the municipality |  |  |  |  |  |
| 2.4. | A mayor is above all an initiator, a coordinator, shaping relations, cooperation and networks in city management |  |  |  |  |  |
| 2.5 | A mayor should be first and foremost guided by the values of common interest and solidarity in the management of the municipality |  |  |  |  |  |

Part 3

| Type of municipality | |
| --- | --- |
| rural |  |
| urban-rural |  |
| urban |  |

| Administration size | |
| --- | --- |
| < 30 employees |  |
| 31 - 100 employees |  |
| > 100 employees |  |

| DATA OF THE HOLDER OF THE OFFICE OF MAYOR | |
| --- | --- |
| Number of terms as mayor |  |
| Seniority in public administration (years) |  |
| Employment in the business sector (YES/NO) |  |
| Self-employment (YES/NO) |  |
| Do you belong to a political party (YES/NO) |  |
| Are you a member of a non-governmental organisation? (YES/NO) |  |

# Questionnaire (native version: Polish language)

Część 1 Kultura organizacyjna gmin

- 1. PROSZĘ SCHARAKTERYZOWAĆ KULTURĘ ORGANIZACYJNĄ PANA/I URZĘDU ROZDZIELAJĄC WEDŁUG UZNANIA 100 PKT POMIĘDZY 4 PONIŻSZE STWIERDZENIA. IM BARDZIEJ ZGADZA SIĘ PAN/PANI Z DANYM STWIERDZENIEM, TYM WIĘCEJ PUNKTÓW POWINNO BYĆ MU PRZYPISANE. NAJWIĘCEJ PUNKTÓW NALEŻY PRZYPISAĆ ODPOWIEDZI, KTÓRA JEST NAJBLIŻSZA SYTUACJI W PANA/PANI URZĘDZIE. Proszę posługiwać się punktacją 0, 5, 10, 15, 20, 25, …., 90, 95, 100). Przyznane punkty muszą sumować się do 100.

|  | Urząd jest miejscem osobistego spotkania, przyjemnym miejscem pracy. Przypomina wielką rodzinę. Pracownicy mocno się angażują |
| --- | --- |
|  | W urzędzie liczą się postawy proinnowacyjne, energia i przedsiębiorczość, ludzie chętnie ryzykują |
|  | W urzędzie liczą się przede wszystkim wyniki. Główną troską jest najlepsze wykonanie zadań. Pracownicy są ambitni i nastawieni na osiągnięcia |
|  | W urzędzie obowiązuje ścisła hierarchia i kontrola. Tym, co robią ludzie, zazwyczaj rządzą formalne procedury |
| 100 | OGÓŁEM |

- 1. PROSZĘ SCHARAKTERYZOWAĆ PRZYWÓDZTWO W PANA/I URZĘDZIE ROZDZIELAJĄC WEDŁUG UZNANIA 100 PKT POMIĘDZY 4 PONIŻSZE STWIERDZENIA. IM BARDZIEJ ZGADZA SIĘ PAN/PANI Z DANYM STWIERDZENIEM, TYM WIĘCEJ PUNKTÓW POWINNO BYĆ MU PRZYPISANE. NAJWIĘCEJ PUNKTÓW NALEŻY PRZYPISAĆ ODPOWIEDZI, KTÓRA JEST NAJBLIŻSZA SYTUACJI W PANA/PANI URZĘDZIE. Proszę posługiwać się punktacją 0, 5, 10, 15, 20, 25, …., 90, 95, 100). Przyznane punkty muszą sumować się do 100.

|  | Przywództwo w urzędzie powszechnie utożsamia się ze służeniem radą i pomocą oraz roztaczaniem opieki |
| --- | --- |
|  | Przywództwo w urzędzie powszechnie utożsamia się z przedsiębiorczością, nowatorstwem i podejmowaniem ryzyka |
|  | Przywództwo w urzędzie powszechnie utożsamia się ze stanowczością, wysokimi wymaganiami i orientacją na wyniki |
|  | Przywództwo w urzędzie powszechnie utożsamia się z koordynowaniem, sprawnym organizowaniem, stwarzaniem warunków do osiągania dobrych wyników |
| 100 | OGÓŁEM |

- 1. PROSZĘ SCHARAKTERYZOWAĆ STYL ZARZĄDZANIA PRACOWNIKAMI W PANA/I URZĘDZIE ROZDZIELAJĄC WEDŁUG UZNANIA 100 PKT POMIĘDZY 4 PONIŻSZE STWIERDZENIA. IM BARDZIEJ ZGADZA SIĘ PAN/PANI Z DANYM STWIERDZENIEM, TYM WIĘCEJ PUNKTÓW POWINNO BYĆ MU PRZYPISANE. NAJWIĘCEJ PUNKTÓW NALEŻY PRZYPISAĆ ODPOWIEDZI, KTÓRA JEST NAJBLIŻSZA SYTUACJI W PANA/PANI URZĘDZIE. Proszę posługiwać się punktacją 0, 5, 10, 15, 20, 25, …., 90, 95, 100). Przyznane punkty muszą sumować się do 100.

|  | W urzędzie preferuje się pracę zespołową, dąży do powszechnej zgody i uczestnictwa |
| --- | --- |
|  | W urzędzie preferuje się samodzielne podejmowanie ryzyka, innowacyjność, swobodę i oryginalność |
|  | W urzędzie preferuje się ostrą rywalizację, stawiane są wysokie wymagania i liczą się przede wszystkim osiągnięcia |
|  | W urzędzie preferuje się bezpieczeństwo zatrudnienia, podporządkowanie, przewidywalność i niezmienność stosunków |
| 100 | OGÓŁEM |

- 1. PROSZĘ SCHARAKTERYZOWAĆ SPÓJNOŚĆ PANA/I URZĘDU ROZDZIELAJĄC WEDŁUG UZNANIA 100 PKT POMIĘDZY 4 PONIŻSZE STWIERDZENIA. IM BARDZIEJ ZGADZA SIĘ PAN/PANI Z DANYM STWIERDZENIEM, TYM WIĘCEJ PUNKTÓW POWINNO BYĆ MU PRZYPISANE. NAJWIĘCEJ PUNKTÓW NALEŻY PRZYPISAĆ ODPOWIEDZI, KTÓRA JEST NAJBLIŻSZA SYTUACJI W PANA/PANI URZĘDZIE. Proszę posługiwać się punktacją 0, 5, 10, 15, 20, 25, …., 90, 95, 100). Przyznane punkty muszą sumować się do 100.

|  | Spójność urzędu jest zapewniona przez lojalność i wzajemne zaufanie. Wysoko ceni się zaangażowanie w sprawy urzędu |
| --- | --- |
|  | Spójność urzędu jest zapewniona przez zaangażowanie w innowacje i rozwój. Kładzie się nacisk na szukanie nowych dróg i rozwiązań |
|  | Spójność urzędu jest zapewniona przez nacisk na wyniki i osiąganie celów. Powszechnymi motywami działania są ekspansywność i chęć zwyciężenia |
|  | Spójność urzędu jest zapewniona przez formalne zasady i regulaminy. Najważniejsze jest sprawne funkcjonowanie |
| 100 | OGÓŁEM |

- 1. PROSZĘ SCHARAKTERYZOWAĆ REGUŁĘ ZARZĄDZANIA W PANA/I URZĘDZIE ROZDZIELAJĄC WEDŁUG UZNANIA 100 PKT POMIĘDZY 4 PONIŻSZE STWIERDZENIA. IM BARDZIEJ ZGADZA SIĘ PAN/PANI Z DANYM STWIERDZENIEM, TYM WIĘCEJ PUNKTÓW POWINNO BYĆ MU PRZYPISANE. NAJWIĘCEJ PUNKTÓW NALEŻY PRZYPISAĆ ODPOWIEDZI, KTÓRA JEST NAJBLIŻSZA SYTUACJI W PANA/PANI URZĘDZIE. Proszę posługiwać się punktacją 0, 5, 10, 15, 20, 25, …., 90, 95, 100). Przyznane punkty muszą sumować się do 100.

|  | W urzędzie kładzie się nacisk na rozwój osobisty i uczestnictwo. Obserwuje się duże zaufanie, otwartość i współuczestnictwo |
| --- | --- |
|  | W urzędzie kładzie się nacisk na zdobywanie nowych zasobów i podejmowanie nowych wyzwań, eksperymentowanie. Ceni się szukanie nowatorskich rozwiązań i możliwości |
|  | W urzędzie kładzie się nacisk na działania konkurencyjne i wyniki. Liczy się osiąganie ambitnych celów i zwyciężanie |
|  | W urzędzie kładzie się nacisk na trwałość i niezmienność. Ważne są sprawność, kontrola i praca bez zakłóceń |
| 100 | OGÓŁEM |

- 1. PROSZĘ SCHARAKTERYZOWAĆ MIARĘ SUKCESU W PANA/I URZĘDZIE ROZDZIELAJĄC WEDŁUG UZNANIA 100 PKT POMIĘDZY 4 PONIŻSZE STWIERDZENIA. IM BARDZIEJ ZGADZA SIĘ PAN/PANI Z DANYM STWIERDZENIEM, TYM WIĘCEJ PUNKTÓW POWINNO BYĆ MU PRZYPISANE. NAJWIĘCEJ PUNKTÓW NALEŻY PRZYPISAĆ ODPOWIEDZI, KTÓRA JEST NAJBLIŻSZA SYTUACJI W PANA/PANI URZĘDZIE. Proszę posługiwać się punktacją 0, 5, 10, 15, 20, 25, …., 90, 95, 100). Przyznane punkty muszą sumować się do 100.

|  | Za miarę sukcesu uważa się rozwój zasobów ludzkich, pracę zespołową, zaangażowanie pracowników i troskę o ludzi |
| --- | --- |
|  | Za miarę sukcesu uważa się wytwarzanie najbardziej oryginalnych i nowatorskich rozwiązań oraz osiągnięcie pozycji lidera w dziedzinie innowacyjności |
|  | Za miarę sukcesu uważa się pokonywanie konkurentów. Najważniejsze jest osiągnięcie pozycji lidera w urzędzie |
|  | Za miarę sukcesu uważa się sprawność działania. Najważniejsze są: pewność dostaw usług publicznych, dotrzymywanie harmonogramów i niskie koszty |
| 100 | OGÓŁEM |

Część 2 Preferowany styl przywództwa burmistrza

1. CZY PAN/PANI ZGADZA SIĘ LUB NIE ZGADZA Z NASTĘPUJĄCYMI STWIERDZENIAMI?

|  | Stwierdzenia | Zdecydowanie się nie zgadzam | Nie zgadzam się | Ani się nie zgadzam ani się zgadzam | Zgadzam się | Zdecydowanie zgadzam się |
| --- | --- | --- | --- | --- | --- | --- |
| 1.9.3 | Burmistrz/prezydent to przede wszystkim sprawny polityk realizujący założenia programowe swojego ugrupowania |  |  |  |  |  |
| 1.9.4 | Burmistrz/prezydent to przede wszystkim wizjoner i strateg, uwzględniający długofalową perspektywę w zarządzaniu gminą |  |  |  |  |  |
| 1.9.5 | Burmistrz/prezydent to przede wszystkim sprawny menedżer zarządzający gminą |  |  |  |  |  |
| 1.9.6 | Burmistrz/prezydent to przede wszystkim inicjator, koordynator, kształtujący relacje, współpracę i sieci w zarządzaniu miastem |  |  |  |  |  |
| 1.9.7 | Burmistrz/prezydent powinien przede wszystkim kierować się wartościami interesu wspólnego i solidaryzmu w zarządzaniu gminą |  |  |  |  |  |

Część 3 Metryczka

| TYP GMINY | |
| --- | --- |
| Gmina wiejska |  |
| Gmina miejsko-wiejska |  |
| Gmina miejska |  |

| Zatrudnienie w urzędzie | |
| --- | --- |
| Mniej niż 30 osób |  |
| Od 31 do 100 osób |  |
| Powyżej 100 osób |  |

| DANE OSOBY PEŁNIĄCEJ FUNKCJĘ ORGANU WYKONAWCZEGO | |
| --- | --- |
| Liczba kadencji na stanowisku burmistrza/prezydenta miasta |  |
| Okres zatrudnienia w administracji samorządowej (w latach) |  |
| Zatrudnienie w sektorze przedsiębiorstw (tak/nie) |  |
| Prowadzenie własnej działalności gospodarczej (tak/nie) |  |
| Czy przynależy Pan/Pani do partii politycznej? (tak/nie) |  |
| Czy jest Pan/Pani członkiem organizacji pozarządowej? (tak/nie) |  |
